# Acute poisoning related to the recreational use of prescription drugs: an observational study from Oslo, Norway

**DOI:** 10.1186/s12873-019-0271-0

**Published:** 2019-10-15

**Authors:** Marit Mæhle Grimsrud, Mette Brekke, Victoria Lykke Syse, Odd Martin Vallersnes

**Affiliations:** 1 Faculty of Medicine, University of Oslo, Oslo, Norway; 2 The Norwegian PSC Research Center, Oslo University Hospital, Oslo, Norway; 3 General Practice Research Unit, University of Oslo, Oslo, Norway; 4 Oslo Accident and Emergency Outpatient Clinic, Department of Emergency General Practice, City of Oslo Health Agency, Oslo, Norway; 5 Department of General Practice, University of Oslo, Oslo, Norway

**Keywords:** Poisoning, Intoxication, Recreational drug use, Prescription drugs, Benzodiazepines, Opioids

## Abstract

**Background:**

Recreational use of prescription drugs is widespread. We describe acute poisonings related to the recreational use of prescription drugs.

**Methods:**

Retrospective observational study. We retrospectively registered all patients presenting from October 2013 through March 2015 at a primary care emergency outpatient clinic in Oslo, Norway, with an acute poisoning related to recreational drug use. We registered demographic data, toxic agents taken, clinical course and treatment. From this data set we extracted the 819/2218 (36.9%) cases involving one or more prescription drugs.

**Results:**

Among the 819 included cases, 190 (23.2%) were female. Median age was 37 years. The drugs most commonly involved were benzodiazepines in 696 (85.0%) cases, methadone in 60 (7.3%), buprenorphine in 53 (6.5%), other opioids in 56 (6.8%), zopiclone/zolpidem in 26 (3.2%), and methylphenidate in 11 (1.3%). Prescription drugs were combined with other toxic agents in 659 (80.5%) cases; heroin in 351 (42.9%), ethanol in 232 (28.3%), amphetamine in 141 (17.2%), cannabis in 70 (8.5%), gamma-hydroxybutyrate (GHB) in 34 (4.2%), cocaine in 29 (3.5%), and other illegal drugs in 46 (5.6%). The patient was given naloxone in 133 (16.2%) cases, sedation in 15 (1.8%), and flumazenil in 3 (0.4%). In 157 (19.2%) cases, the patient was sent on to hospital.

**Conclusions:**

One in three acute poisonings related to recreational drug use involved prescription drugs. Benzodiazepines were by far the most common class of drugs. Prescription drugs had mostly been taken in combination with illegal drugs or ethanol.

## Introduction

Several classes of prescription drugs may be used for recreational purposes and have the potential to induce tolerance and addiction. Benzodiazepines and opioids are currently the major classes of recreationally used prescription drugs [1–3]. Barbiturates, previously much prescribed, have been more or less replaced by benzodiazepines since the 1980s [4, 5]. Central stimulant drugs prescribed for hyperkinetic disorders may also be used for recreational purposes [6]. Recently, reports have emerged on the recreational use of pregabalin, gabapentin and quetiapine [7–9].

The recent rise in opioid deaths in the USA has been ascribed to an increase in opioid prescription [1]. In the UK primary care setting, there has also been an escalation of prescription of opioids between 2000 and 2010 [10]. Though heroin still is the opioid most frequently involved in fatal overdoses in Europe, the proportion of fatalities involving methadone, buprenorphine, fentanyl and tramadol is rising in several countries [2]. In Norway, the number of fatal opioid overdoses has been stable at 250–300 per year for the last 15 years, but the proportion caused by prescription opioids has increased from 30 to 50% [11].

Data on presentations to emergency departments due to the recreational use of prescription drugs are sparse [12]. Though benzodiazepines and zopiclone/zolpidem (Z-drugs) are reported to constitute 18–57% of acute poisonings treated in European emergency departments, most of these cases were suicide attempts [13–18]. Data for the less frequently appearing prescription drugs have rarely been reported, except in a previous study from Oslo encompassing nearly 3000 cases, where methadone had been taken in 2 % of all poisonings, and buprenorphine in 1 % [15]. Furthermore, in a study of recreational drug toxicity at 16 European centres, benzodiazepines were reported in 20% of cases, methadone in 4 %, buprenorphine in 2 %, and ketamine in 2 % [19].

Some patients are prescribed potentially addictive substances by their doctor and then develop an addiction. Others already have substance use problems and take prescribed drugs as a supplement to illegal drugs. Combination of different types of drugs increases the risk of toxicity. When there is a potential for recreational use, legally bought drugs prescribed by a doctor are also traded on the illegal market. However, a large part of the prescription drugs in circulation on the illegal market in Norway is probably imported illegally from abroad [20]. Benzodiazepines constitute the substance of abuse seized third most frequently by the Norwegian police, after cannabis and amphetamines [3]. A total of 1,152,931 benzodiazepine tablets were seized in 2013 [3]. Since 2010 a large part of the benzodiazepine tablets seized are produced in eastern European countries and smuggled into Norway [3]. Nonetheless, prescription patterns also seem to impact on excessive use and overdoses [1, 21, 22]. Hence, knowing which drugs that show up as toxic agents in emergency departments is an important part of the risk assessment when prescribing.

### Objectives

We describe acute poisonings related to the recreational use of prescription drugs; the drugs taken, combinations with other drugs, the clinical state of the patients, and treatment given.

## Methods

### Design

A retrospective observational study of acute recreational drug toxicity at a primary care emergency outpatient clinic in Oslo, Norway. We used the inclusion criteria and dataset developed by the European Drug Emergencies Network (Euro-DEN) [19, 23].

### Setting

The Norwegian health care system is public and two-tiered. Hospitals and specialist health care services are run by the state, while primary care is organised by the municipalities. There is a strong gate-keeping function. Patients cannot present to hospitals or secondary care specialists directly, but have to be referred by their general practitioner or by a doctor at a primary care emergency outpatient clinic, or triaged for hospital treatment by the ambulance service.

Data was collected from 1 October 2013 through 31 March 2015 (18 months) at the Oslo Accident and Emergencies Outpatient Clinic (OAEOC), the major primary care emergency outpatient clinic in Oslo, Norway. The OAEOC covers the entire city of Oslo at all hours (population 647,676 as per 1 January 2015 [24]). There are about 200,000 consultations per year. Patients with acute recreational drug toxicity can be observed locally for 4 h [25]. Diagnostic resources and treatment options are limited, and patients in need of more intensive observation or treatment are sent on to hospital. Toxicological laboratory tests to determine toxic agents are not used. The majority of patients with acute recreational drug toxicity in Oslo are treated at the OAEOC, though the more severely poisoned patients are brought directly to hospitals by the ambulance service [15, 25].

### Participants

All patients presenting to the OAEOC with symptoms or signs related to acute recreational drug toxicity were included. A recreational drug was defined as any psychoactive substance taken for recreational purposes. Classification of recreational use was based on the assessment made by the doctor treating the patient, as noted in the electronic medical records. Patients who had been poisoned against their will, or who had taken a toxic agent for purposes of self-harm, were not included. Patients with poisoning from alcohol only were not included.

Eligible patients were identified retrospectively from the patient registration lists in the local electronic medical records. Inclusion was based on the information in these records, as noted by the doctor treating the patient. Each presentation was registered as a unique case, and we did not trace patients to see whether they presented to the OAEOC more than once.

For this study, we extracted the cases with one or more prescription drugs among the recreational drugs taken.

### Data collection and classification

Data was collected from the local electronic medical records and from local observational charts.

We registered age, gender, toxic agents taken, time of presentation, whether the patient was brought by ambulance, vital signs at presentation (respiratory rate, heart rate, and Glasgow Coma Scale (GCS) score), clinical features during the course of the poisoning episode (hypertension (systolic blood pressure ≥ 180 mmHg), hypotension (systolic blood pressure ≤ 90 mmHg), hyperthermia (temperature ≥ 39 °C), vomiting, headache, anxiety, hallucinations, agitation, psychosis, seizures, palpitations, chest pain, and arrhythmias), length of stay, treatment given, and disposition (death, admitted somatic hospital, admitted psychiatric ward, medically discharged, or self-discharge).

Toxic agents were determined based on the assessment done by the doctor treating the patient, as noted in the electronic medical records. The doctors’ assessments were based on information from the patient and/or the patient’s companions, and on the signs and symptoms seen. Toxicological laboratory tests were not done. The registration of clinical features was also based on the assessment done by the doctor treating the patient. Though the doctors at the OAEOC did not include patients and register data, they were familiar with the research protocol. They are also locally trained to treat poisoned patients, including assessment of intention and toxic agents.

We categorised all benzodiazepines together, but separately from the Z-drugs. When categorising opioids, we kept methadone and buprenorphine separate, as they are the two drugs used in the Norwegian opioid substitution treatment program. Furthermore, we categorised the rest of the opioids according to the Norwegian prescription regulations, where most opioids are subject to the strictest rules (class A). Codeine, tramadol and ethylmorphine are less strictly regulated (class B) than the other opioids, though still subject to stricter rules than ordinary prescription drugs (class C).

For comparisons across age, we made the following categories: ≤ 19 years, 20–29 years, 30–39 years, 40–49 years, 50–59 years, ≥ 60 years.

### Statistical analyses

All analyses were done in IBM SPSS version 25. We used Mann-Whitney U-test for comparing age between genders. As more than one toxic agent was taken in many cases, one case may be counted in several categories of prescription drugs. Hence, there are overlaps between the categories, and they are not independent of each other for statistical purposes. Consequently, mere descriptions are presented, using percentages for categorical variables and medians and interquartile ranges for continuous variables.

When converting the continuous variables respiratory rate and heart rate into the categorical variables bradypnoea (respiratory rate < 10 per minute), tachycardia (heart rate > 99 per minute) and bradycardia (heart rate < 50 per minute), missing data was categorised as the clinical feature not being present. Otherwise, missing data were kept out of the analyses.

### Ethics

The study was part of a quality improvement study. It was approved by the director of the Department of Emergency General Practice at the City of Oslo Health Agency, and by the Oslo University Hospital Information Security and Privacy Office (ref no 2013/3706).

## Results

There were 2218 cases of acute poisoning related to recreational drug use during the 18 months of inclusion. In 819 (36.9%) cases the patient had taken a prescription drug and was included in this study. Among the 819 included cases, 190 (23.2%) were female. Median age was 37 years (interquartile range (IQR) 28–47). There was no significant difference in age between genders (*p* = 0.11).

Benzodiazepines were the most frequent prescription drugs taken, in 696 (85.0%) cases, followed by methadone in 60 (7.3%) cases and buprenorphine in 53 (6.5%) cases (Table 1). In 174 (21.2%) cases, more than one prescription drug was taken.

**Table 1:** Recreationally used prescription drugs in acute poisoning during 18 months at a primary care emergency outpatient clinic in Oslo, Norway

Drug	*n*	(%)
Benzodiazepines	696	(85.0)
*Clonazepam*	411	(50.2)
*Diazepam*	110	(13.4)
*Alprazolam*	80	(9.8)
*Oxazepam*	64	(7.8)
*Flunitrazepam*	23	(2.8)
*Nitrazepam*	16	(2.0)
*Flurazepam*	1	(0.1)
*Unspecified*	73	(8.9)
Methadone	60	(7.3)
Buprenorphine	53	(6.5)
Opioids class A^a^	35	(4.3)
*Morphine*	25	(3.1)
*Oxycodone*	9	(1.1)
*Pethidine*	1	(0.1)
Z-drugs	26	(3.2)
*Zopiclone*	20	(2.4)
*Zolpidem*	6	(0.7)
Opioids class B^b^	22	(2.7)
*Codeine*	15	(1.8)
*Tramadol*	7	(0.9)
*Ethylmorphine*	1	(0.1)
Methylphenidate	11	(1.3)
Pregabalin	6	(0.7)
Quetiapine	6	(0.7)
Others	6	(0.7)
*Ketamine*	3	(0.4)
*Gabapentin*	2	(0.2)
*Modafinil*	1	(0.1)
Total	819	(100)

More than one prescription drug was taken in 174 (21.2%) of cases. Hence, sums of fractions are higher than total

^a^Class A is the strictest regimen of prescription by Norwegian regulations. It applies to nearly all opioids, methylphenidate, and ketamine

^b^Prescription of class B drugs is less strictly regulated than class A, but more than ordinary prescription drugs (class C). It applies to benzodiazepines, Z-drugs, some opioids, pregabalin, and modafinil

Prescription drugs were combined with other toxic agents in 659 (80.5%) cases (Table 2); illegal drugs in 525 (64.1%) cases and ethanol in 232 (28.3%). More specifically, prescription drugs were combined with heroin in 351 (42.9%) cases, amphetamine in 141 (17.2%), cannabis in 70 (8.5%), gamma-hydroxybutyrate (GHB) in 34 (4.2%), cocaine in 29 (3.5%), and other illegal drugs in 46 (5.6%).

**Table 2:** Combinations of prescription drugs and other drugs in acute poisoning related to recreational drug use

	Benzodiazepines n (%)	Z-drugs n (%)	Methadone n (%)	Buprenorphine n (%)	Opioids class A^a^ n (%)	Opioids class B^b^ n (%)	Methylphenidate n (%)	Pregabalin n (%)	Quetiapine n (%)	Others n (%)
Benzodiazepines	NA	10 (38.5)	30 (50.0)	28 (52.8)	14 (40.0)	5 (22.7)	2 (18.2)	3 (50.0)	1 (16.7)	3 (50.0)
Prescription opioids^c^	74 (10.6)	4 (15.4)	NA	NA	NA	NA	1 (9.1)	–	–	1 (16.7)
Other prescription drugs^d^	19 (2.7)	NA	–	2 (3.8)	2 (5.7)	2 (9.1.)	NA	NA	NA	NA
Heroin	329 (47.3)	3 (11.5)	13 (21.7)	9 (17.0)	14 (40.0)	4 (18.2)	–	4 (66.7)	–	2 (33.3)
Amphetamine	129 (18.5)	–	4 (6.7)	11 (20.8)	4 (11.4)	–	2 (18.2)	2 (33.3)	–	2 (33.3)
Cocaine	22 (3.2)	–	–	–	–	4 (18.2)	–	–	2 (33.3)	1 (16.7)
GHB	33 (4.7)	–	1 (1.7)	–	–	1 (4.5)	–	–	–	–
Cannabis	63 (9.1)	1 (3.8)	3 (5.0)	5 (9.4)	3 (8.6)	3 (13.6)	1 (9.1)	–	1 (16.7)	1 (16.7)
Other illegal drugs^e^	42 (6.0)	–	–	2 (3.8)	–	2 (9.1)	1 (9.1)	–	–	–
Any illegal drug	476 (68.4)	3 (11.5)	19 (31.7)	23 (43.4)	21 (60.0)	13 (59.1)	4 (36.4)	5 (83.3)	2 (33.3)	3 (50.0)
Alcohol	202 (29.0)	11 (42.3)	5 (8.3)	12 (22.6)	7 (20.0)	6 (27.3)	3 (27.3)	2 (33.3)	2 (33.3)	1 (16.7)
Total	696 (100)	26 (100)	60 (100)	53 (100)	35 (100)	22 (100)	11 (100)	6 (100)	6 (100)	6 (100)

*NA* Not applicable

Percentages are proportions of cases each prescription drug category (columns) combined with the specified drugs (rows). As many drugs were combined in some cases, and only one drug taken in other cases, percentages do not add up to total

^a^Class A is the strictest regimen of prescription by Norwegian regulations

^b^Prescription of class B drugs is less strictly regulated than class A, but more than ordinary prescription drugs (class C)

^c^Methadone, buprenorphine, opioids class A and opioids class B

^d^Z-drugs, methylphenidate, pregabalin, quetiapine, others

^e^Including 27 cases of unspecified opioids

GHB: Gamma-hydroxybutyrate

The patient was brought by ambulance in 449 (54.8%) cases (Table 3). The patient presented at night (22:00–05:59) in 257 (31.4%) cases, and during the weekend in 221 (27.0%) cases. Median length of stay was 4 h 31 min (IQR 2 h 31 min – 6 h 21 min). In 183 (22.3%) cases, the patient was given treatment other than mere observation; 133 (16.2%) were given naloxone, 15 (1.8%) were sedated, and three (0.4%) were given flumazenil. From the outpatient clinic, 143 (17.5%) were sent on to somatic hospital, 14 (1.7%) were admitted to a psychiatric ward, 526 (64.2%) were medically discharged, and 136 (16.6%) self-discharged. No patients died at the outpatient clinic.

**Table 3:** Demographic data and clinical course for patients presenting with acute poisoning related to recreational drug use

	Benzodiazepines n (%)	Z-drugs n (%)	Methadone n (%)	Buprenorphine n (%)	Opioids class A^a^ n (%)	Opioids class B^b^ n (%)	Methylphenidate n (%)	Pregabalin n (%)	Quetiapine n (%)	Others n (%)	All cases^c^ n (%)
Gender											
*Females*	162 (23.3)	13 (50.0)	12 (20.0)	12 (22.6)	5 (14.3)	7 (31.8)	3 (27.3)	4 (66.7)	4 (66.7)	2 (33.3)	190 (23.2)
*Males*	534 (76.7)	13 (50.0)	48 (80.0)	41 (77.4)	30 (85.7)	15 (68.2)	8 (72.7)	2 (33.3)	2 (33.3)	4 (66.7)	629 (76.8)
Age^d,e^	35 (28–46)	49 (32–61)	44 (36–50)	37 (30–49)	46 (33–54)	37 (31–46)	28 (23–39)	37 (33–48)	29 (19–37)	40 (24–45)	37 (28–47)
Brought by ambulance	375 (53.9)	12 (46.2)	34 (56.7)	28 (52.8)	24 (68.6)	16 (72.7)	6 (54.5)	3 (50.0)	5 (83.3)	4 (66.7)	449 (54.8)
Time of presentation											
*Night*^*f*^	222 (31.9)	11 (42.3)	15 (25.0)	13 (24.5)	8 (22.9)	5 (22.7)	5 (45.5)	1 (16.7)	3 (50.0)	2 (33.3)	257 (31.4)
*Weekend*^*g*^	185 (26.6)	10 (38.5)	11 (18.3)	17 (32.1)	8 (22.9)	6 (27.3)	4 (36.4)	5 (83.3)	3 (50.0)	1 (16.7)	221 (27.0)
Length of stay^d,h^	4:39 (2:41–6:29)	3:12 (2:01–4:07)	4:39 (2:37–6:47)	4:28 (2:30–6:55)	3:19 (2:15–4:42)	2:35 (1:42–4:49)	2:00 (1:16–4:42)	3:01 (1:54–4:45)	2:44 (1:34–6:16)	2:29 (2:09–3:26)	4:31 (2:31–6:21)
Treatment^i^	154 (22.1)	7 (26.9)	16 (26.7)	6 (11.3)	9 (25.7)	7 (31.8)	3 (27.3)	1 (16.7)	–	1 (16.7)	183 (22.3)
*Naloxone*	117 (16.8)	4 (15.4)	11 (18.3)	4 (7.5)	8 (22.9)	5 (22.7)	1 (9.1)	–	–	1 (16.7)	133 (16.2)
*Flumazenil*	2 (0.3)	–	–	1 (1.9)	–	–	–	–	–	–	3 (0.4)
*Sedation*	11 (1.6)	1 (3.8)	–	–	–	1 (4.5)	2 (18.2)	–	–	1 (16.7)	15 (1.8)
Disposition											
*Admitted somatic hospital*	118 (17.0)	8 (30.8)	18 (30.0)	5 (9.4)	7 (20.0)	5 (22.7)	3 (27.3)	1 (16.7)	–	3 (50.0)	143 (17.5)
*Admitted psychiatric ward*	9 (1.3)	2 (7.7)	1 (1.7)	1 (1.9)	1 (2.9)	–	2 (18.2)	–	1 (16.7)	–	14 (1.7)
*Medically discharged*	454 (65.2)	13 (50.0)	34 (56.7)	37 (69.8)	20 (57.1)	10 (45.5)	4 (36.4)	5 (83.3)	4 (66.7)	3 (50.0)	526 (64.2)
*Self-discharge*	115 (16.5)	3 (11.5)	7 (11.7)	10 (18.9)	7 (20.0)	7 (31.8)	2 (18.2)	–	1 (16.7)	–	136 (16.6)
Total	696 (100)	26 (100)	60 (100)	53 (100)	35 (100)	22 (100)	11 (100)	6 (100)	6 (100)	6 (100)	819 (100)

^a^Class A is the strictest regimen of prescription by Norwegian regulations

^b^Prescription of class B drugs is less strictly regulated than class A, but more than ordinary prescription drugs (class C)

^c^As many patients had taken more than one drug, the numbers for the separate prescription drug categories add up to more than the total number of cases

^d^Median (interquartile range)

^e^Missing data in 5 cases

^f^Night: 22:00–05:59

^g^Saturdays and Sundays

^h^In hours:minutes

^i^Any treatment other than mere observation

Benzodiazepines were the most frequently reported drugs in all age groups. Patients reporting methylphenidate or quetiapine were younger than the others, median age 28 years (IQR 23–39) and 29 years (IQR 19–37) respectively, compared to 37 years (IQR 28–47) in the total material. Patients reporting Z-drugs, opioids class A, and methadone were older, median age 49 years (IQR 32–61), 46 years (IQR 33–54), and 44 years (IQR 36–50), respectively (Table 3).

Lowered conscious level was the most common clinical feature; 536 (65.3%) had a GCS score of 14 or less, and 24 (2.9%) were in a coma with GCS score of 7 or less (Table 4). Furthermore, 185 (22.6%) were tachycardic and 84 (10.3%) bradypnoeic at presentation, while 95 (11.6%) were agitated at some point of time.

**Table 4:** Clinical state of patients presenting with acute poisoning related to recreational drug use

	Benzodiazepines n (%)	Z-drugs n (%)	Methadone n (%)	Buprenorphine n (%)	Opioids class A^a^ n (%)	Opioids class B^b^ n (%)	Methylphenidate n (%)	Pregabalin n (%)	Quetiapine n (%)	Others n (%)	All cases^c^ n (%)
Bradypnoea											
*(RR < 10)*^*d*^	79 (11.4)	2 (7.7)	5 (8.3)	3 (5.7)	5 (14.3)	–	–	–	–	–	84 (10.3)
Tachycardia											
*(HR > 99)*^*d*^	155 (22.3)	4 (15.4)	10 (16.7)	11 (20.8)	10 (28.6)	6 (27.3)	6 (54.5)	1 (16.7)	2 (33.3)	3 (50.0)	185 (22.6)
Bradycardia											
*(HR < 50)*^*d*^	13 (1.9)	–	–	1 (1.9)	–	–	–	–	–	–	13 (1.6)
Hypertension											
*(SBP ≥ 180)*	3 (0.4)	–	1 (1.7)	–	–	–	–	–	–	–	3 (0.4)
Hypotension											
*(SBP ≤ 90)*	29 (4.2)	–	3 (5.0)	4 (7.5)	3 (8.6)	–	–	–	1 (16.7)	–	36 (4.4)
Hyperthermia											
*(tp ≥ 39.0)*	4 (0.6)	–	1 (1.7)	–	–	–	–	–	–	1 (16.7)	5 (0.6)
GCS score^d,e^											
*15*	231 (33.2)	16 (61.5)	13 (21.7)	14 (26.4)	13 (37.1)	17 (77.3)	6 (66.7)	3 (50.0)	6 (100)	5 (83.3)	283 (34.6)
*8–14*	444 (63.8)	10 (38.5)	43 (71.7)	39 (73.6)	21 (60.0)	4 (18.2)	3 (33.3)	2 (33.3)	–	1 (16.7)	509 (62.1)
*3–7*	20 (2.9)	–	4 (6.7)	–	1 (2.9)	1 (4.5)	–	1 (16.7)	–	–	24 (2.9)
Vomiting	12 (1.7)	1 (3.8)	5 (8.3)	3 (5.7)	1 (2.9)	2 (9.1)	1 (9.1)	–	–	1 (16.7)	22 (2.7)
Headache	15 (2.2)	2 (7.7)	1 (1.7)	2 (3.8)	1 (2.9)	1 (4.5)	–	1 (16.7)	–	1 (16.7)	19 (2.3)
Anxiety	17 (2.4)	1 (3.8)	1 (1.7)	3 (5.7)	1 (2.9)	2 (9.1)	2 (18.2)	–	1 (16.7)	2 (33.3)	26 (3.2)
Hallucinations	8 (1.1)	2 (7.7)	2 (3.3)	3 (5.7)	2 (5.7)	–	2 (18.2)	–	–	–	16 (2.0)
Agitation	77 (11.1)	1 (3.8)	10 (16.7)	9 (17.0)	6 (17.1)	–	5 (45.5)	–	–	1 (16.7)	95 (11.6)
Psychosis	12 (1.7)	2 (7.7)	2 (3.3)	1 (1.9)	3 (8.6)	1 (4.5)	4 (36.4)	–	–	–	21 (2.6)
Seizures	4 (0.6)	–	–	–	–	–	–	–	–	–	4 (0.5)
Palpitations	1 (0.1)	–	–	–	–	1 (4.5)	1 (9.1)	–	3 (50.0)	2 (33.3)	7 (0.9)
Chest pain	11 (1.6)	–	–	–	2 (5.7)	1 (4.5)	–	–	1 (16.7)	–	14 (1.7)
Arrhythmias	2 (0.3)	1 (3.8)	–	–	–	–	–	–	–	–	3 (0.4)
Total	696 (100)	26 (100)	60 (100)	53 (100)	35 (100)	22 (100)	11 (100)	6 (100)	6 (100)	6 (100)	819 (100)

*GCS* Glasgow Coma Scale; *HR* heart rate per minute; *RR* respiratory rate per minute; *SBP* systolic blood pressure in mmHg; *tp* temperature in °Celsius

^a^Class A is the strictest regimen of prescription by Norwegian regulations

^b^Prescription of class B drugs is less strictly regulated than class A, but more than ordinary prescription drugs (class C)

^c^As many patients had taken more than one drug, the numbers for the separate prescription drug categories may add up to more than the total number of cases

^d^On presentation

^e^Missing data in 3 cases

## Discussion

### Summary of main findings

Prescription drugs had been taken in one out of three poisonings related to recreational drug use. Benzodiazepines were by far the most common class of drugs, reported in 85% of the cases involving prescription drugs, followed by the substitution program opioids methadone and buprenorphine, reported in 7 % and 6 % of the cases, respectively. In two out of three cases prescription drugs were combined with illegal drugs, and in one out of three cases with ethanol. One in five needed treatment other than mere observation, and one in five were sent on to hospital.

### Benzodiazepines

The predominance of benzodiazepines is consistent with previous European studies of acute poisoning related to recreational drug use [13, 15, 19], and with data on drugs seized by the Norwegian police [3]. Compared with previous studies, there has been a gradual increase in the number of benzodiazepine and Z-drug cases per year at the OAEOC, amounting to a 35% increase since 2008 [14, 15]. Benzodiazepines were nearly always combined with other drugs, most frequently heroin, but also with amphetamine. Prescription opioids were also frequently combined with benzodiazepines. In studies substantiating patient reports with laboratory testing of toxic agents, benzodiazepines show up in even more cases, and often combined with both heroin and amphetamine [26, 27]. Benzodiazepines may enhance the euphoric effects of other recreational drugs, take the edge off unwanted side effects, and alleviate withdrawal symptoms [28–30]. This may explain the extensive co-use of benzodiazepines and other drugs. This co-use, however, is not without risk, as benzodiazepines may potentiate the respiratory depression caused by opioids and other central depressants [31].

### Opioids

Prescription opioids were the second most frequent class of prescription drug reported, also in line with previous European studies [13, 15, 19]. The availability of prescription opioids has been increasing both in the USA [1] and the UK [10]. However, this is not the case in Norway, where the number of defined daily doses of both class A and class B opioid pain killers sold from wholesalers is decreasing [32]. In our study, the numbers of cases with methadone and buprenorphine per year at the OAEOC were in the same range as in a previous study from 2012 [15], but doubled compared to 2008 [14]. The number of patients in opioid substitution programs in Norway increased from about 2500 in 2003 to 7000 in 2013 [33]. Diversion of opioids from opioid substitution programs does occur, and in our study methadone and buprenorphine were reported in more than twice as many cases as other prescription opioids. This is also in line with misused methadone and buprenorphine being the two most commonly used opioids in Europe after heroin, and the increasing number of deaths from these opioids in Norway [2, 11]. However, the harms of diverted methadone and buprenorphine must be weighed against the clearly demonstrated benefits of opioid substitution programs [34–37]. Opioid diversion is probably an unavoidable side effect of these programs.

### Other drugs

In addition to benzodiazepines, Z-drugs and opioids, smaller numbers of other prescription drugs also appeared; methylphenidate, a central stimulant used to treat attention deficit hyperactivity disorder (ADHD); pregabalin and gabapentin, used for neuralgia and as antiepileptics; quetiapine, an antipsychotic and antidepressant; ketamine, a dissociative anaesthetic; and modafinil, used to treat narcolepsy. Recreational use of all these substances has been previously reported [6–9, 38–40]. There were only three cases of ketamine in our study, while in a European multi-centre study of recreational drug toxicity ketamine was reported in 2.3% of cases [19], possibly indicating that ketamine is not much used for recreational purposes in Norway.

### Clinical course

Clinical features were as would be expected from the agents taken. Depressant effects predominated, the stimulant effects of methylphenidate constituting a notable exception. Though no more than one in five needed treatment beyond mere observation, the need for observation is obvious since two out of three had a conscious level of 14 or less, and one out of ten were agitated. Furthermore, 19.2% were sent on to hospital, needing more intensive observation and/or treatment than available at the outpatient clinic. This is slightly less than the 23.4% (519/2218) among all cases treated for acute recreational drug toxicity at the OAEOC during the inclusion period [41].

### Strengths and limitations

The majority of patients treated for acute recreational drug toxicity in Oslo are treated at the OAEOC. Hence, our study encompasses most patients treated for acute poisoning related to the recreational use of prescription drugs in a European capital city. However, patients in a more severe clinical state are transported directly to hospital by the ambulance service [25]. Therefore, the clinical state seen in our data may not be representative, underestimating the range of severity of poisoning with the reported agents.

No laboratory tests were used to verify the toxic agents. Registration of toxic agents was based on the assessment of the doctor treating the patient, again much based on the patient’s report of agents taken. However, the assessments were made in real clinical situations, and decisions on patient management were based on them. In studies comparing laboratory testing with clinical assessments, patient reports of drugs taken are often found to be correct, though incomplete [26, 27]. Hence, our study probably underestimates the number of drugs taken, and some cases related to recreational use of prescription drugs were probably missed.

We did not register information on the source of the prescription drugs taken. Accordingly, we do not know whether our patients had taken drugs actually prescribed by their own doctor, by somebody else’s doctor, or acquired on the illegal market.

In some categories the numbers are small, and the results must be interpreted with care.

## Conclusions

Prescription drugs, mainly benzodiazepines and mainly combined with illegal drugs or ethanol, appeared in one out of three cases of acute poisoning related to recreational drug use. Thus, prescription drugs contribute significantly to the pool of toxic agents showing up in these poisonings. Often taken in combination with other drugs, potentiating their effects, prescription drugs also contribute to the severity of the poisonings. Clinicians should be aware of the panorama of multi-drug use. Though we do not know to which extent the prescription drugs in our study were actually prescribed or acquired on the illegal market, caution is called for when prescribing drugs with a potential for recreational use. A strict prescription policy may reduce the availability of drugs for diversion to the illegal market, and may reduce the risk of creating iatrogenic drug addiction.

## Data Availability

Data are currently not available for sharing. Several manuscripts based on the data set are in preparation. Requests concerning the data may be sent to the corresponding author.

## Abbreviations

GCS: Glasgow Coma Scale
GHB: Gamma-hydroxybutyrate
HR: Heart rate
IQR: Interquartile range
NA: Not applicable
OAEOC: Oslo Accident and Emergency Outpatient Clinic
RR: Respiratory rate
SPB: Systolic blood pressure
tp: Temperature
Z-drugs: Zolpidem and zopiclone
